# Mapping Consistent Rice (*Oryza sativa* L.) Yield QTLs under Drought Stress in Target Rainfed Environments

**DOI:** 10.1186/s12284-015-0053-6

**Published:** 2015-07-24

**Authors:** Silvas J Prince, R Beena, S Michael Gomez, S Senthivel, R Chandra Babu

**Affiliations:** 1 Centre for Plant Molecular Biology and Biotechnology, Tamil Nadu Agricultural University (TNAU), Coimbatore, 641 003 India; 2 International Center for Tropical Agriculture (CIAT), Colombia, 6713 Colombia; 3 Agricultural Research Station, Tamil Nadu Agricultural University (TNAU), Paramakudi, 623707 India

**Keywords:** Rice, Rainfed ecosystem, Drought resistance, Yield under stress, Secondary traits, Quantitative trait locus, Marker-assisted breeding

## Abstract

**Background:**

Drought stress is a major limitation to rainfed rice production and yield stability. Identifying yield-associated quantitative trait loci (QTLs) that are consistent under drought stress predominant in target production environments, as well as across different genetic backgrounds, will help to develop high-yielding rice cultivars suitable for water-limited environments through marker-assisted breeding (MAB). Considerable progress has been made in mapping QTLs for drought resistance traits in rice; however, few have been successfully used in MAB.

**Results:**

Recombinant inbred lines of IR20 × Nootripathu, two *indica* cultivars adapted to rainfed target populations of environments (TPEs), were evaluated in one and two seasons under managed stress and in a rainfed target drought stress environment, respectively. In the managed stress environment, the severity of the stress meant that measurements could be made only on secondary traits and biomass. In the target environment, the lines experienced varying timings, durations, and intensities of drought stress. The rice recombinant inbred lines exhibited significant genotypic variation for physio-morphological, phenological, and plant production traits under drought. Nine and 24 QTLs for physio-morphological and plant production traits were identified in managed and natural drought stress conditions in the TPEs, respectively. Yield QTLs that were consistent in the target environment over seasons were identified on chromosomes 1, 4, and 6, which could stabilize the productivity in high-yielding rice lines in a water-limited rainfed ecosystem. These yield QTLs also govern highly heritable key secondary traits, such as leaf drying, canopy temperature, panicle harvest index and harvest index.

**Conclusion:**

Three QTL regions on chromosome 1 (RM8085), chromosome 4 (I12S), and chromosome 6 (RM6836) harbor significant additive QTLs for various physiological and yield traits under drought stress. The similar chromosomal region on 4 and 6 were found to harbor QTLs for canopy temperature and leaf drying under drought stress conditions. Thus, the identified large effect yield QTLs could be introgressed to develop rice lines with stable yields under varying natural drought stress predominant in TPEs.

**Electronic supplementary material:**

The online version of this article (doi:10.1186/s12284-015-0053-6) contains supplementary material, which is available to authorized users.

## Background

Globally, rice is grown on 154 million hectares (Mha), and approximately 45 % of this area is under rainfed conditions that have very low-yield potential (Verulkar et al. 2010). Rainfed rice are grown in 60 Mha of land area (Fischer et al. 2012). In Asia, drought stress is the most pervasive threat to both rainfed lowland (46 Mha) and upland (10 Mha) rice production, affecting the yield stability (Pandey et al. 2007). In Tamil Nadu, in the southern part of India, rice is predominantly grown under rainfed condition during north-east monsoon season (September–December). During this season, drought stress occurs during both vegetative and reproductive stages. The present drought study was conducted in this rainfed target environment situated at 9 °N latitude and 78 °E longitude, with an average seasonal rainfall of only 475 mm during this cropping period (based on 50 years of data). Even in traditionally irrigated areas, which accounts for almost 75 % of total rice production, drought is becoming an increasing problem because of water scarcity, which has resulted from a rising demand for water for competing uses (Fischer et al. 2012). Thus, developing drought-resistant rice cultivars is important to reduce climate-related risk, to increase productivity, and to alleviate poverty among rainfed farmers (Venuprasad et al. 2008).

Direct selection for yield under stress in managed stress environments (MSEs) (Venuprasad et al. 2007) and target environments (TEs) (Kumar et al. 2008; Yadaw et al. 2013) is considered a promising approach to improve drought tolerance in rice. However, direct selection for yield under drought in TEs is difficult because of differences in the timing and severity of drought over seasons. Hence, identifying secondary traits contributing to drought resistance may improve selection efficiency. Atlin and Lafitte (2002) reported certain secondary traits that correlated with yield under stress; however, with little proven success (Kumar et al. 2008). In drying soils, secondary traits, such as green leaf area or canopy temperature, could be used effectively to screen huge numbers of genotypes (Richards et al. 2010). However, incorporation of secondary trait(s) as a selection criterion in breeding is hampered by complex phenotypic protocols. Alternatively, quantitative trait locus (QTL) mapping followed by marker-assisted breeding (MAB) could be an effective approach to identify genomic regions linked to crop performance in stressful environments, and pyramiding the desirable alleles could improve drought resistance in crops (Ashraf, 2010). In the last 20 years, considerable progress has been made towards mapping QTLs for drought resistance traits in rice (Kamoshita et al. 2008); however, there have been few successful cases of their application in MAB (Steele et al. 2009). The success rate of using QTLs in molecular breeding reflects the lack of repeatability of QTL effects across genetic backgrounds and environments (Bernier et al. 2008).

In recent years, several researchers developed mapping populations between high-yielding lines (IR64, Swarna and MTU1010) and drought-tolerant local landraces and wild cultivars to map grain yield QTLs (Srividhya et al. 2011; Vikram et al. 2011; Ghimire et al. 2012; Yadaw et al. 2013) for reproductive stage-specific drought stress. To the best our knowledge, none of the studies were conducted under natural drought conditions predominant in TEs and these QTLs were identified in MSE and QTLs mapped under severe drought stress conditions (Kumar et al. 2008). Successful marker-assisted selection to improve yield mainly relied on the use of high-yielding lines to identify large-effect QTLs (Vikram et al. 2011) and evaluation of their consistent effects in TEs (Yadaw et al. 2013). Recently, Weber et al. (2012) also showed less correlation between managed and random drought stress environments for grain yield in maize. Studies in MSE may limit the chances of detecting QTLs for drought resistance that are widely applicable to target populations of environments (TPEs), as the timing and intensity of stress vary over years in rainfed rice ecosystems (Pandey et al. 2007), which ultimately changes the plants’ responses and traits involved in drought-resistance mechanisms (Kamoshita et al. 2008). A TPE is the set of all environments, farms, and future seasons in which an improved variety will be grown (IRRI International Rice Research Institute 2006; Fischer et al. 2012). Most of the *indica* × *indica* derived rice lines used in QTL mapping of drought resistance were not adapted to TPEs (Ali et al. 2000; Kamoshita et al. 2002; Manickavelu et al. 2006; Biji et al. 2008). Serraj et al. (2011) also emphasized the importance of field experiments in TPEs to identify QTLs for rice yield under natural drought stress. Earlier, Gomez et al. (2010) used recombinant inbred lines (RILs) derived from locally adapted *indica* rice lines to detect QTLs for plant production traits under drought stress in TPEs, but no yield QTL was identified. The present study was conducted with the objectives: (1) To map consistent large-effect yield QTLs in a mapping population developed by crossing a high-yielding rice line, IR20, and a landrace, Nootripathu (NP), under natural drought predominant in the TPE; and (2) to identify key secondary traits associated with grain yield in MSE and TE.

## Results

### Effect of Drought Stress in MSE and TPE

**Fig. 1:**
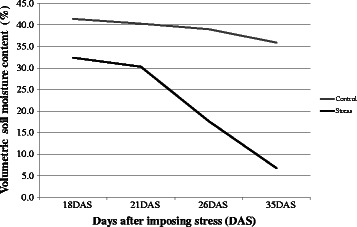
Depletion of soil moisture content (%) in stress and control plots in MSE (Trial 1)

**Fig. 2:**
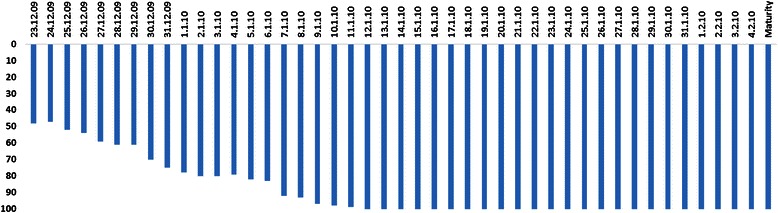
Depletion of soil moisture (in cm) from 82 days after emergence to maturity under rainfed condition in Trial 3

Considerable variations in the timing of drought stress (the crop growth stage that experienced the drought stress), duration, and intensity were observed in both managed and target environment trials (Table 1). The experimental plots of Trial 1 experienced severe drought stress conditions, with high evaporative demand. During this season, the crop lacked irrigation: the water source irrigation well dried completely because of the severe dry season. Thus, even the control treatment plots could not be irrigated during the panicle initiation stage. However, the depletion of the soil moisture content (%) in the stress plots was high compared with the irrigated control plots (Additional file 1: Table S1) after 18–35 days of stress imposition (Fig. 1). The RILs did not flower, even in control plots, and biomass was the only measure of plant production measured in this trial. On average, the RILs recorded 50.2 % reduction in biomass under water stress and showed significance at all levels (Table 2). In trial 2 of the TPE, the RILs under rainfed treatment experienced a dry spell of 26 days during flowering, which resulted in a reduction of grain yield by 38.7 % and straw yield by 27.9 %. Trial 3 experienced a dry spell for 16 days at the grain-filling stage, and depletion of the soil water table was evident from 77 days after emergence in the drought stress plots. The soil water table depleted to 100 cm at the grain-filling phase (102 days after emergence) and declined further until maturity (Fig. 2); its effect was pronounced, with significant reductions in spikelet fertility.

**Table 1:** Site, soil, and drought characterization of field trials conducted in a managed water environment (trial 1 in Coimbatore) and in TPE (trials 2–3 in Paramakudi) India

Characteristics	Trial 1 2004	Trial 2 2004	Trial 3 2009
Elevation above MSL (m)	427	40	40
Latitude	11° 59, 43,, N	9° 54, 59,, N	9° 33, 03,, N
Longitude	77° 34, 57,, E	70° 34, 57,, E	70° 34, 57,, E
Soil texture	Clay	Clay	Clay
Soil pH	8.4	8.1	8.1
Timing of start of stress (days after emergence)	87	62	84
Total duration of stress period (days)	36	26	16
Rainfall during stress period (mm)	No rainfall	No rainfall	3
Number and duration of continuous rain free days during stress period	1 (22 days)	1 (26 days)	1 (16 days)
Rainfall during crop period (mm)	312	621	486
Maximum Temperature (°C)	31.5	32.1	40.0
Minimum Temperature (°C)	20.0	20.3	26.0
Average relative humidity (%)	81.2	87.1	86.4

**Table 2:** Trait mean and range values for 200 recombinant inbred lines and their parental lines tested under drought stress in MSE during 2004 (trial 1)

Traits	IR20	Nootripathu	Mean	Range	S.D.	H	LSD _(*α* = 0.05)_	Significance		
								Genotype (G)	Treatment (T)	G X T
Leaf rolling-Stress	7.0	5.0	6.5	5.0–7.0	0.60	0.63	1.8168	0.0279	-	-
Leaf drying-Stress	6.0	6.0	4.9	2.0–7.0	0.87	0.72	2.1746	<.0001	-	-
Stress recovery	4.3	5.7	5.8	1.7–7.0	0.91	0.70	1.9855	<.0001	-	-
Canopy temperature (°C) Stress	40.6	38.4	40.1	27.8–43.5	1.54	0.86	3.4874	0.1081	-	-
SPAD value Stress	20.5	38.1	30.9	20.0–39.5	4.00	0.62	0.4189	<.0001	<.0001	<.0001
Irrigated	32.0	38.0	33.3	21.1–42.5	4.10	0.74				
Plantheight (cm) Stress	33.2	45.7	42.5	28.3–69.0	7.50	0.48	1.0589	<.0001	<.0001	0.0663
Irrigated	38.6	50.8	51.4	32.3–86.2	10.70	0.63				
Tiller number Stress	3.0	5.6	4.9	2.8–11.3	1.70	0.36	0.2313	<.0001	<.0001	0.9079
Irrigated	3.8	5.8	5.5	2.5–18.2	1.70	0.42				
Biomass (g m^−2^) Stress	108.0	226.0	194.0	804.4–1020.8	91.10	0.63	13.299	<.0001	<.0001	<.0001
Irrigated	206.3	412.5	389.5	140.0–1343.0	165.70	0.68

### Variation of Physio-morphological, Phenology, and Production Traits

Significant variation was observed among the RILs and parents for plant phenology and production traits in the MSE (Table 2) and TPE (Additional file 2: Table S2). The RILs transgressed the parents for the measured traits and showed a normal distribution. In trial 1 (MSE), the drought tolerant parent, NP, recorded higher leaf chlorophyll (SPAD), maintained a cooler canopy temperature (CT) under drought stress, and showed better drought stress recovery compared with IR20 (Table 2). It also showed a higher yield than IR20 under severe drought stress. Plant height was positively correlated, while canopy temperature and leaf drying were negatively correlated with biomass under stress in trial 1 in the MSE (Additional file 3: Table S3). In the MSE, most of the traits measured showed a higher significance at genotype levels (with low significance for leaf rolling (LR) and canopy temperature) and treatment levels (production traits alone). Canopy temperature showed a higher heritability than biomass and other physiological traits measured under the MSE, and was positively correlated with parameters of water stress indicators; i.e., leaf rolling and leaf drying, LD (Additional file 3: Table S3). The genetic relationship between leaf drying (−0.12) and canopy temperature (−0.15) was negative with grain yield under stress conditions.

In the TPE, the grain yield under non-stress conditions had a moderate to high *H* value, ranging from 0.34 to 0.70 in trials 2 and 3, and a low to high *H* value under stress conditions (Additional file 2: Table S2). In the TPE, significant positive correlations were observed among plant height, panicle length, number of productive tillers, panicle harvest index (PHI), and spikelet fertility and grain yield under stress. Days to 50 % flowering was negatively correlated with grain yield under stress in the TPE (Additional file 4: Table S4). The grain yield and harvest index, HI (measured in TE) shared a positive and significant genetic relationship (results not shown). Interestingly, another secondary trait, PHI, also showed a significant positive correlation with the HI. However, the HI was positively correlated with grain yield under stress, measured within each environment only. The panicle HI showed significant linkage in all trials.

### QTLs Mapped in the TE and MSE

The linkage map used in the present study was an updated version of the map constructed earlier, with 105 marker loci comprising a map length of 1532 cM, with an average distance of 14.6 cM between any two marker loci. Inclusion of additional markers in the map reduced the linkage groups from 17 to 12 in this study. Putative main effect QTLs identified under stress conditions in each of the test environments are given in Table 3. QTL analysis detected nine major QTLs explaining a phenotypic variation ranging from 11 to 36.8 % for the plant water relations and production traits measured in the MSE (Table 3). Twenty-four QTLs were identified with phenotypic variation ranging from 4.3 to 55.8 % for the various phenology and plant production traits under drought stress measured from the two (trials 2 and 3) TPE experiments (Table 3). The QTL, RM314 on chromosome 6 explained the highest phenotypic variation of 55.8 % for days to flowering under drought stress in trial 2 of the TPE. Similarly, QTL RM8085 on chromosome 1 explained the highest phenotypic variations of 52.2 and 20.9 % for plant height and grain yield, respectively under drought stress in the TPE in trial 3. Interestingly, major QTLs for grain yield under drought stress in the TPE co-located at these chromosomal regions; i.e., RM8085 on chromosome 1 in trial 3 (20.9 %) and at RM314 on chromosome 6 in trial 2 (14.0 %) of the TPE. Major QTLs for HI (44.9 %), panicle HI (24.5 %) and 100-seed weight (36.1 %) also overlapped at RM314 in chromosome 6 in trial 2 of the TPE. Another QTL region, near marker C20 on chromosome 4, was linked to biomass under severe drought stress in the MSE, explaining 36.8 % of the phenotypic variation. Biomass was the only measure of plant production in this trial. An adjacent QTL, I12S on chromosome 4, was detected for grain yield under drought stress in trial 3 of the TPE, explaining 19.6 % of the phenotypic variation.

**Table 3:** QTLs detected for physio-morphological and plant production traits under drought stress condition in MSE (trial1) and TE (trials 2 and 3)

Trait^a^	Trial	Chr.	Nearest marker	Position (cM)	LOD score	R^2^ (%)	Additive effect^a^
Relative water content (%)	1	8	RM6925	6.26	3.60	11.0	0.12
Canopy temperature (°C)	1	7	RM3691	44.54	2.60	11.0	0.67
Leaf rolling	1	6	RM314	21.88	4.20	24.8	−0.29
		12	RM101	68.73	5.23	27.3	−0.33
Stress recovery	1	2	RM208	126.35	3.30	18.4	0.40
Days to 50 % flowering	2	6	RM314	21.85	30.0	55.8	−5.01
Plant height (cm)	1	1	RM212	126.40	3.40	20.0	0.41
		2	RM2770	0.00	3.00	15.0	0.34
		9	RM6862	186.38	3.27	13.2	0.23
	2	1	RM212	126.40	14.2	27.5	12.2
		8	RM1235	6.28	2.53	5.0	4.93
	3	1	RM8085	247.8	12.48	52.2	11.56
Tiller number	2	2	A11	0.35	2.61	4.8	0.55
			C06M1	62.94	3.00	6.8	−0.33
Productive tillers	2	2	C06M1	62.92	5.30	10.0	−0.29
		7	RM6449	18.14	2.52	5.60	−0.23
		10	RM1859	0.30	3.01	6.0	−0.23
Panicle length (cm)	2	1	RM212	126.37	5.42	10.7	0.81
		8	RM1235	0.00	2.52	4.3	0.50
Panicle HI	2	6	RM314	21.87	11.0	24.5	0.06
100 Seed weight (g)	2	2	RM1342	78.27	3.2	5.9	0.06
		6	RM314	21.85	18.3	36.1	0.35
Grain yield (g/m^2^)	2	6	RM314	21.88	7.00	14.0	26.0
		8	RM6925	6.26	3.50	8.5	24.5
	3	1	RM8085	241.8	6.53	20.9	19.36
		6	RM6836	37.9	3.99	6.7	11.34
Straw yield (g/m^2^)	2	1	RM9	85.33	3.37	6.3	−47.6
			RM3825	134.00	8.41	17.8	86.1
		6	RM314	21.85	10.0	20.0	−87.8
		6	RM6836	40.81	6.00	9.1	−194.81
	3	6	RM314	32.9	3.1	5.4	−29.89
Biomass (g/m^2^)	1	4	C20	22.23	14.0	36.8	0.06
Harvest index	2	1	RM3825	133.98	2.7	5.0	−0.03
		6	RM314	21.85	25.0	44.9	0.09
	3	6	RM314	30.9	10.36	20.2	0.04

^a^Positive and negative values indicate that the IR20 and NP allele increase the phenotypic value, respectively, for a particular trait

#### QTLs for Leaf Physiological Traits and Yield

A QTL for leaf relative water content under drought stress in the MSE was detected near RM6925 on chromosome 8, which explained 11 % of the phenotypic variation (Table 3). A QTL for grain yield under natural drought in the TPE was located at this interval in trial 2. A QTL for canopy temperature under drought stress in the MSE was identified near RM 3691 on chromosome 7, explaining 11 % of the phenotypic variation (Table 3). A QTL for leaf rolling identified on chromosome 6 explained a phenotypic variation of 24.8 % in the MSE. This region was also found to be associated with days to 50 % flowering, grain yield, straw yield, HI, and PHI under natural drought in trials conducted at the TPE (Table 3). Another QTL for leaf rolling under drought in the MSE was detected near RM101 on chromosome 12, explaining 27.3 % of the phenotypic variation in trial 1. A QTL for stress recovery identified on chromosome 2 explained 18.4 % of the phenotypic variation in the MSE. Region C20 on chromosome 4 was found to be associated with leaf chlorophyll content (SPAD) under drought stress in the MSE in trial 1 (Table 4); this QTL also explained 36.8 % of the phenotypic variation of biomass under stress in MSE in trial 1.

**Table 4:** List of QTLs with additive effects identified under stress conditions over different seasons in the target environment

Trait	Trial	Chr.	Marker Interval	Confidence interval^a^	A ± SE^b^	*α* = 0.05	
						*F* value^c^	h^2^(a)^d^
Canopy temperature	1	4	I12S–P16	91.3–111.3	1.07 ± 0.20	32.9	1.8
		6	RM6836–S12M1	31.5–44.8	−0.76 ± 0.12	17.9	1.4
Leaf drying	1	4	I12S–P16	104.3–116.3	−0.30 ± 0.06	13.87	6.8
SPAD	1	4	RM5424–C20	39.4–69.3	−1.02 ± 0.27	14.11	6.7
Grain yield (g/m^2^)	2	1	RM8085–RM3825	144.7–166.2	−38.68 ± 10.26	14.25	7.2
		4	I12S–P16	80.3–111.3	−70.49 ± 16.38	16.52	8.9
Straw yield (g/m^2^)	2	4	RM6909–I12S	78.3–78.3	−59.90 ± 16.19	13.70	6.4
Panicle harvest index	2	4	I12S–P16	84.3–116.3	−0.15 ± 0.04	13.44	6.5

^a^Confidence interval in CentiMorgans with respect to the first marker in the linkage group

^b^Main additive effect plus/minus standard error. Thus, positive values indicate that the IR20 allele increases the phenotypic value

^c^ *F* value of significance for each QTL

^d^h^2^(a) is the heritability of the additive effect or percentage of variation that is explained by the additive component of the QTL

Consistent QTLs for yield-related traits, i.e., grain yield, straw yield, and HI under drought stress in the TPE, were detected on chromosome 6 near RM314 across experiments conducted over different years. The QTL for grain yield under drought near RM8085 on chromosome 1 explained a higher phenotypic variation of 20.9 % in trial 3 of the TPE. Similarly, a QTL for days to 50 % flowering under drought was identified near RM314 on chromosome 6, explaining 55.8 % of the phenotypic variation in trial 2, with the positive allele coming from the landrace, Nootripathu. The same QTL was also associated with 100-seed weight, grain yield, straw yield, panicle HI, and HI under drought stress in trial 2 in the TPE, with the positive allele inherited from IR20 (Table 3). Another QTL near RM6836 on chromosome 6 was consistently linked to grain and straw yield in trials 2 and 3 in the TPE. These three QTL regions, RM8085 on chromosome 1, I12S on chromosome 4, and RM6836 on chromosome 6 showed significant additive QTLs for various physiological and yield traits under drought stress conditions (Table 4).

### Genes Underlying Candidate QTL Regions

**Fig. 3:**
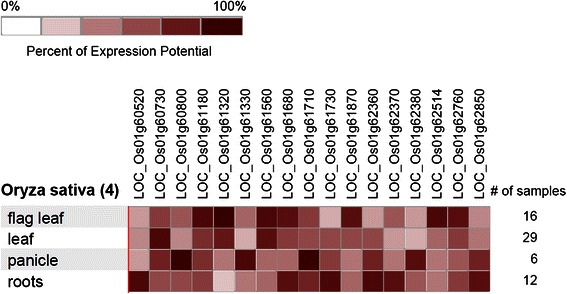
Expression of genes in the QTL region on chromosome 1 under drought stress

**Fig. 4:**
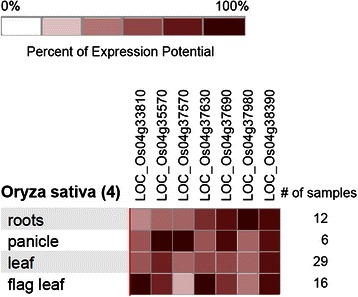
Expression of genes in the QTL region on chromosome 4 under drought stress

The major QTLs identified were mined and found to possess 248 genes in an interval of 1.61Mbp (chromosome 1; RM8085–RM3825), 350 genes in an interval of 2.4Mbp (Chromosome 4; RM5424–RM3042) (Additional file 5: Table S5), two genes (Chromosome 4; RM6909) and 1 gene (Chromosome 6; RM6836). In the chromosome 1 QTL region, 17 genes were highly expressed in drought stress conditions on the flag leaf, leaf, panicle, and root tissues, as shown in Fig. 3. However, only two genes showed high expression in the panicle: unknown expressed protein (*LOC_Os01g60800*) and transport protein, coatomer subunit delta-3 (*LOC_Os01g61710*). A putative thioredoxin (*LOC_Os01g61320*), and WRKY56 transcription factor (*LOC_Os01g62514*) showed high expression in the flag leaf under drought stress. Among the genes identified in the QTL on chromosome 4, seven genes showed high expression in different tissues under drought stress, as shown in Fig. 4. The regulator of chromosome condensation (*LOC_Os04g35570*) and aspartic proteinase nepenthesin (*LOC_Os04g37570*) showed high expression in the panicle and transporter family protein, *LOC_Os04g37980* in roots. An RNA recognition motif containing protein (*LOC_Os04g33810*) and mitochondrial carrier protein (*LOC_Os04g37630*) showed higher expression in flag leaf tissues. The other QTL peak on chromosome 4 near RM6909 positioned near two genes (*LOC_Os04g53510* and *LOC_Os04g53520*) showed moderate levels of expression in drought stress conditions. The chromosome 6 QTL region was located near a gene encoding a Suppressor of variegation 3–9, the Polycomb-group chromatin regulator Enhancer of zeste and the trithorax-group chromatin regulator Trithorax (SET) domain-containing protein, LOC_Os06g16390, which also showed moderate expression in the panicle and roots.

## Discussion

### Understanding Components Underlying Yield under Drought Conditions

Drought stress is the major abiotic stress limiting rice production, especially in rainfed ecosystems. Developing cultivars combining drought resilience and high-yield potential will help to increase rainfed rice production. In both trials in the TE, the HI showed significant association with grain yield under stress. A trait-based approach with precise understanding of the TPE will improve selection efficiency for molecular breeding strategies. Correlating genetic information with physiological traits will also help to develop drought-tolerant rice varieties (Lanceras et al. 2004). In this study, leaf drying and canopy temperature were positively correlated and showed higher heritability in the MSE and TE (Gomez et al. 2010). These secondary traits could also be used as indirect selection indices to select genotypes with better root traits (Lopes and Reynolds 2010) which translates into higher grain yield in TE (Suji et al. 2012 a, b). Under the MSE, canopy temperature was negatively correlated with biomass. A similar relationship was reported previously (Babu et al. 2003) and canopy temperature was also shown to be negatively correlated with spikelet fertility and grain yield under drought stress in rice (Garrity and OToole, 1995).

Even though the rainfall pattern and distribution varied among the trials in the TE, significant variation for grain yield, straw yield, and HI were observed among the RILs. The reduction in grain yield indicated that different stages within the reproductive phase, such as peduncle elongation, anthesis, and fertilization, are critical in determining yield under drought stress. In rice, the flowering period is highly sensitive to water stress, which increases the pollen and spikelet sterility (Jongdee et al. 2002). Interestingly, certain secondary traits, such as panicle HI, which is significantly associated with HI, a trait genetically correlated with yield under stress, might be useful in the selection process. Similar panicle-associated traits (HI and panicle exertion, which also influences panicle HI) are reported to be more reliable for indirect selection of grain yield under stress in both upland and lowland adapted populations (Kumar et al. 2008).

### QTLs for Physio-morphological Traits Influence Yield under Drought Stress

No yield QTLs were identified in the MSE (trial 1) because of drought severity. However, a large-effect biomass QTL was detected on chromosome 4 near marker C20 that explained the highest phenotypic variation (36.8 %; Table 3). Interestingly this region was also linked to leaf chlorophyll content (SPAD). Selection based on chlorophyll content showed a higher relative efficiency than direct selection for yield in maize (Ziyomo and Bernardo 2013). The other yield QTL on chromosome 6 (RM314) also governed a QTL for leaf rolling, days to 50 % flowering, stress recovery, 100-seed weight, leaf rolling, panicle HI, and HI. In addition, this region also influences flowering, with a higher R^2^ value of 55.8 %, so it may enhance yield under drought through early flowering, which is a drought-escape mechanism. Interestingly, the QTL region I12S on chromosome 4 also harbors additive QTLs for canopy temperature, leaf drying, and panicle HI, and might interact with QTL region RM6836-S12M1 on chromosome 6, which affects both grain and straw yield, in addition to affecting canopy temperature. The other large-effect additive QTL identified on chromosome 1 near RM8085 explained a higher proportion of the phenotypic expression, based on the level of drought stress observed in the TE. Similar increases in the expressions of yield QTLs with response to drought stress was reported by Yadaw et al. (2013). The grain yield QTL region on chromosome 2 was reported to contain QTLs for leaf rolling, leaf drying, canopy temperature, productive tiller number, and stress recovery in this mapping population (Gomez et al. 2010). This same region was reported to contain QTLs for panicle number under stress in a Vandana/Way Rarem population of rice (Bernier et al. 2007). Thus, the documentation of physiological phenotypes other than yield parameters could permit progress in breeding and developing higher-yielding crops in stress environments (Tardieu and Tuberosa, 2010). In addition the identified genomic regions associated with yield under stress in this study interact with key physiological/secondary traits, which would result in a yield benefit under drought in the TE.

### Yield QTLs under Drought Stress

Among yield QTLs identified in the TE, two genomic regions on chromosome 1 (RM8085), and 6 (RM314) showed larger effects (14.0 and 20.9 % of the phenotypic variation with the positive allele from the drought-sensitive parent, IR20). Thus, these QTL regions represent interesting genetic regions for further investigation to confirm that the susceptible genotypes contribute superior alleles for yield under stress (Lafitte et al. 2004 a, b). There are several examples wherein positive alleles for grain yield under drought stress were contributed by the drought-susceptible parents (Lanceras et al. 2004; Bernier et al. 2007). Previously, a meta-QTL analysis revealed the presence of yield QTLs on chromosome 1 (Vikram et al. 2011) in almost 50 % of 92 drought panel rice lines, which included donors such as traditional landraces. QTL interaction analysis also identified similar regions on chromosomes 1, 4, and 6 (Table 4) explaining a phenotypic variation from 1.4 to 8.9 %, with significant F values. Interestingly, the QTL for yield under stress near RM8085 on chromosome 1 was consistent across the QTL analysis in the TPE, explaining higher levels of phenotypic variation. Maccaferri et al. (2008) also emphasized the consistent expression of a QTL across a broad range of agro-meteorological conditions and that the coincidence of QTLs across environments (Cattivelli et al. 2008) is critical to breed crops for wide adaptation and yield stability. The region RM314 on chromosome 6 is associated with yield and yield-related traits (straw yield and HI) and also showed a large effect of phenotypic variation. These QTL regions on chromosome 1 and 6 are associated with various physio-morphological and plant production traits under drought stress in rice (Gomez et al. 2010; Kanagaraj et al. 2010; Salunkhe et al. 2011).

Among the three yield-associated meta-QTLs identified on chromosome 1, based on a genome-wide analysis, the region RM543–RM212 spans a small genetic distance of 0.27 kb and makes it suitable for use in MAB and pyramiding of QTLs for yield and drought tolerance in rice (Swamy et al. 2011). Thus, this large-effect QTL region could be directly used to develop high-yielding lines for the TPE without further validation. Another significant additive QTL detected on chromosome 6 (RM6836) was linked to yield-related traits and HI under stress conditions. However, this region was linked with grain yield only in trial 3, because the crop experienced drought at a later stage of grain filling in the TE. Similar QTLs with large effects on grain yield and/or flowering unique to particular hydrological conditions were reported previously by several other researchers (Bernier et al. 2007; Kumar et al. 2007; Venuprasad et al. 2009). The allele for the grain yield QTL in this region was inherited from the landrace, Nootripathu. Similarly, a QTL (on RM217) linked with grain yield under stress was reported near RM314 on chromosome 6, with the allele inherited from another rice landrace, Norungan, which is also adapted to this TPE (Suji et al. 2012a). This QTL region was also associated with PSII maximum efficiency and explained 12.9 % of the phenotypic variance under stress during grain filling stage in rice (Gu et al. 2011). Thus, these two QTL regions on chromosome 1 (RM212–RM8085) and chromosome 6 (RM314–RM6836) were consistent across environments (trials) for plant height, panicle length, straw yield, and HI under stress. In addition, they have additive effects on grain and straw yield under stress conditions. Thus, selecting these positive alleles with stable effects in the mixture of drought scenarios encountered in the TPE may help developing rice cultivars for drought-prone environments (Tardieu, 2012).

### Co-location of Yield Components and Candidate Genes Underlying Yield QTLs

The RM212 region on chromosome 1 also showed higher phenotypic variation for shoot biomass at flowering and HI under stress (Kumar et al. 2007). This region comprises *short panicle1* (sp1) and *LAX PANICLE 1* (*LAX1*) genes, which regulate the number of spikelets per panicle by enhancing meristematic activity and promoting cell proliferation (Xing and Zhang, 2010). A nearby simple sequence repeat marker, RM443, co-segregated with a pollen sterility QTL in *O.sativa*/*O.glaberrima* lines (Li et al. 2008). This QTL region was reported to harbor genes involved in cellular metabolism, transport and signal transduction, transcription, and hormonal regulation (Pradeepa et al. 2012). Lenka et al. (2011) identified the expression of major genes for 4,5 DOPA dioxygenase extradiol, glycosyltransferases, amino acid transporters, MADS-box family gene, and serine/threonine protein kinases under drought conditions in this QTL region. Swamy et al. (2011) identified genes encoding a pentatricopeptide repeat protein and a leucine zipper protein in this region, which govern flowering and restore fertility in rice. The role of the four novel genes that are expressed at higher levels in the panicle and flag leaf tissues on chromosome 1 require functional validation for their association with grain yield under stress. On chromosome 6, a grain weight QTL was mapped near RM6836 and narrowed down to 4.7 cM (Guo et al. 2006) in an *indica*/*japonica* mapping population. Bian et al. (2010) reported that this region harbors QTLs for 1000 grain weight, grain length, and grain width in chromosome substitution lines developed between *indica/japonica* rice lines. Ebana et al. (2011) reported that this region is associated with heading date in cultivated rice with a higher phenotypic variation of 70 %. It is also possible that these genes that confer a grain yield advantage under stress may have undergone strong natural selection to stay together and remain conserved during the course of evolution. A SET domain-containing protein involved in the methylation process was observed to be moderately expressed in the panicle and roots in this region. This could be a candidate gene that modulates the root and shoot response to drought to ensure yield under stress conditions.

### Epistatic Interaction of QTLs for Secondary Traits and Yield under Stress

The secondary traits, such as canopy temperature (loci on chromosomes 4 and 6) and leaf drying (loci on chromosome 4), co-locate with yield QTLs under stress (Table 4). Under drought stress, lower canopy temperature indicates favorable plant water status and it also acts as a drought avoidance mechanism (Jones et al. 2009). The region RM314 on chromosome 6 explained a higher proportion of phenotypic variation for both leaf rolling and grain yield in drought stress conditions. In the rainfed TPE, leaf drying scores could be correlated to grain yield under stress in rice (Lafitte et al. 2004a). Recently, leaf drying was also reported as a reliable criterion for indirect selection in maize (Ziyomo and Bernardo, 2013) to improve yield under drought-prone environments (Haider et al. 2012). Epistatic QTL interactions were reported for canopy temperature, leaf water potential, and spikelet fertility in a Zhenshan97B/IRAT109 rice mapping population (Liu et al. 2005). The co-location of these QTLs and the phenotypic correlations among them reflect the existence of genetic relationships between the physiological traits, canopy temperature and leaf drying, and grain yield under drought stress in rice. Thus, understanding the key physiological mechanism responsible for drought resistance, and the identification of alleles that could be applied in breeding, will hasten the development of drought adaptive cultivars (Sellamuthu et al. 2011). Thus, with a few high-yielding popular varieties occupying a large area in the drought-prone rainfed ecosystem, identifying major QTLs with consistent effects across the background of popular variety, IR20, and introgression into same/other drought-susceptible varieties, could be an effective strategy for MAB (Serraj et al. 2011; Ghimire et al. 2012). The consistent large-effect QTLs identified for yield that interact with key physiological/secondary traits under stress conditions in the TPE represent a unique opportunity for breeders to introgress them into other high-yielding drought-susceptible varieties through MAB (Dixit et al. 2012).

## Conclusion

The yield QTLs identified in the present study are consistent and proved to be effective under varying levels of drought stress predominant in the TE. The putative QTLs identified on chromosomes 1, 4, and 6 are key targets to enhance productivity in the rainfed rice ecosystem, through direct selection for grain yield and also to harness the benefits of underlying key secondary traits. The secondary trait, HI, which is significantly related to grain yield under stress could be used as an indirect selection index in the TE. In addition, these yield-related QTLs identified in the TE could be directly used to develop high-yielding rice lines suitable for rainfed rice ecosystems, without further validation or testing. Thus, the introgression of these key yield QTLs will help rainfed farmers to obtain high and stable yields under the natural drought stress that is predominant in TE.

## Materials and Methods

### Mapping Population

IR20 is a popular *indica* cultivar that is highly sensitive to drought, with shallow and thin roots (Babu et al. 2001). It is a semi-dwarf variety with profuse tillers and high yield, suitable for irrigated conditions. Despite its drought sensitivity, it is grown considerably under rainfed conditions in southern Tamil Nadu State, India, because of its grain yield, quality and marketability. Nootripathu is a drought-resistant *indica* landrace from the rainfed rice ecosystem of Tamil Nadu, India, which has deep and thick roots (Babu et al. 2001). It is a tall plant with few tillers, low-yield potential, and poor grain quality. Three hundred and ninety-seven RILs were developed from a cross between IR20 and Nootripathu. From the 397 F_7_ RILs, a subset of 200 F_8_ lines was evaluated for physio-morphological and production traits under MSE during the dry season (February–May, 2004) in the experimental fields of the University at Coimbatore, India (Trial 1). Another subset of 340 F_8_ lines (Trial 2) was evaluated under rainfed conditions in the TPE in the experimental fields of the Agricultural Research Station of the University at Paramakudi, India during 2004. Further, a subset of 330 F_11_ RILs was tested under rainfed conditions in the same TPE during 2009 (Trial 3). The details on the experimental locations and their site characteristics are given in Table 1.

### Field Experiments

#### Managed Stress Environment

In trial 1 (MSE), the RILs and their parents were evaluated in replicated plots in a randomized complete block design during the dry season of 2004. The lines were planted in plots of 2.0 × 0.4 m^2^ with a spacing of 20 × 10 cm between and within rows, respectively, both in irrigated (two replications) and water stress (three replications) conditions. The experimental plots were surface irrigated once every 4 days to field capacity. At the panicle initiation stage (80 days after sowing), irrigation was withheld in stress plots to impose drought stress. Physio-morphological measurements were made during peak stress, after the RILs showed leaf rolling and drying symptoms. LR and LD scores were recorded three times during the stress period, based on a 1–9 scale standardized for rice (IRRI International Rice Research Institute 1996) and average values were derived. At midday, CT was recorded using an infrared thermometer (AG-42, Teletemp Corporation, CA, USA) with an 8° field of view and equipped with a 10.5- to 12.5-μm band pass filter, as described by Garrity and O’ Toole (1995). The measurement was made at noon by facing south to minimize the effects of sunlight. Leaf chlorophyll content was determined in the second youngest fully expanded leaf, using a handheld SPAD meter (SPAD 502, Minolta Camera Co. NJ, USA). The chlorophyll content was presented as SPAD readings (Hua et al. 2006). At maturity, plant height and number of productive tillers were averaged based on three randomly selected plants and straw yields were recorded in all the RILs and parents on a whole plot basis.

#### Target Population of Environments

In the TPE, two trials were conducted from September to December during 2004 (trial 2) and 2009 (trial 3) under natural drought stress conditions during the northeast monsoon (wet) seasons. The lines were grown in three replicates under irrigated (non-stress) and rainfed (natural drought stress) conditions in plots of 2.0 × 0.4 m^2^ (trial 2) and 2.5 × 0.2 m^2^ (trial 3). The seeds were sown in dry soil at a seed rate of 80 kg ha^−1^, with a spacing of 20 × 10 cm between and within rows, respectively. Stress plots were completely rainfed from sowing to harvest, and control plots were surface irrigated to field capacity at regular intervals. Data on plant height, number of productive tillers, panicle length, and spikelet fertility (ratio of number of filled grains/total number of grains (filled + unfilled) per panicle expressed as percentages) were measured from three randomly selected hills. Data on days to 50 % flowering, grain yield, and biomass were recorded using all the plants from the whole plot. In addition, the panicle harvest index (PHI) was calculated as the ratio of grain weight of filled grains to total panicle weight for each RIL and 100-seed weight was measured in trial 2. The soil water content was measured using eight peizometers that were installed diagonally across the plots to cover the entire plots of trial 3.

### Statistical Analysis

Statistical analysis was carried out using the SAS statistics package general linear model (GLM) procedure (SAS Institute Inc 1990). The frequency distribution was assessed to test the trait skewness among the RILs. The broad sense heritability *(H)* was calculated from the covariance values using the formula, *H* = σ^2^_G_/(σ^2^_G_+ σ^2^_e_/k), where σ^2^_G_ and σ^2^_e_ are the genetic and residual variances, respectively, and ‘k’ is the number of replications. The required variance components for calculating heritability were obtained as explained by Fehr (1987). The relationship between grain yield under stress and secondary traits was analyzed using linear regression (SPSS statistical package v.21, IBM Corp. Released 2012) considering yield under stress as the fixed effect.

### Genotyping and Molecular Map Construction

A framework genetic map comprising 101 loci, which included 71 simple sequence repeat, 21 random amplified polymorphic DNA, eight inter-simple sequence repeat, and one expressed sequence tag markers was constructed previously in this laboratory using the same subset of 250 F_7_ RILs of this mapping population (Gomez et al. 2010). In the present study, the parents, IR20 and NP, were genotyped with 635 rice microsatellite markers, and 25 polymorphic markers were used in genotyping the mapping progenies. The genotypic data were generated with 250 RI lines and tested for *χ*^2^ goodness of fit against a 1:1 segregation ratio. Among the polymorphic markers, four markers alone segregated in the expected ratio of 1:1 at 0.01 % probability, and were added to the previous linkage map by reconstruction of the map with a logarithm of odds (LOD) of 3.0 and a minimal distance of 50 cM, by Map Manager QTX software (Manly et al. 2001) using the Haldane mapping function.

### QTL Analysis

QTL analysis was performed for each trial individually, using a composite interval mapping (CIM) approach in WINQTLCART v.2.5 software (Basten et al. 2005). Cofactors for this analysis were selected using the forward regression method. In WINQTLCART, model six was selected, with five control marker numbers and a window size of 10 cM. A significance threshold value of 2.5 was determined after 1000 permutations for the traits analyzed. The phenotypic variation explained by a single QTL was calculated as the square of the partial correlation coefficient (partial R^2^) by the final multiple regression model. QTL analyses for phenology and plant production traits were carried out for all the three experiments (trial 1–3), whereas QTL analysis for physio-morphological traits was done only for the experiment conducted in MSE (trial 1).

QTL interactions and their effects were identified using QTLNetwork v2.0 (Yang et al. 2008). To identify significant QTLs and interactions, critical F values for each trait were determined after 1000 permutations. Candidate interval selection, epistatic effects, and putative QTL detection were calculated with an experimental-wide type I error of *α* = 0.05 each. Genome scanning was performed using a 10-cM window size and with a 1-cM walk speed. Phenotypic data from a common subsets of RILs (202 lines) from four individual trials (environments) were combined as input data. Data on canopy temperature, leaf rolling, leaf drying, chlorophyll content, biomass (from Gomez et al. 2010), panicle HI (from trial 2), and grain and straw yield data (from trial 2) under drought stress conditions were used for this analysis.

### Candidate Genes within QTLs Identified and their Expression Pattern

The details for the candidate genes within the identified QTLs were selected based on Nipponbare sequence information (Kawahara et al. 2013). The expression of the genes within the QTL intervals were obtained from drought stress experiment analyses using Affymetrix gene chip data (NCBI database: GSE24048, GSE26280, and GSE25176) available in Genevestigator (Hruz et al. 2008).

## Additional files

Additional file 1: Table S1. Mean value of mineral and organic soil moisture contents of irrigated and water stressed field in trial 1. Additional file 2: Table S2. Trait mean and range values for RILs and parental lines evaluated in TPE during trial 2 and 3. Additional file 3: Table S3 Correlation coefficients among biomass and physio-morphological parameters measured under drought stress in MSE during 2004–2005 (Trial 1). Additional file 4: Table S4 Correlation coefficients among plant phenology and production traits under rainfed conditions in trial 2 (2004–05) and trial 3 (2009–10) conducted in TPE. Additional file 5: Table S5 List of genes identified within QTL identified on chromosme 1 and 4.

## References

[CR1] Ali ML, Pathan MS, Zhang J, Bai G, Sarkarung S, Nguyen HT (2000). Mapping QTLs for root traits in a recombinant inbred population from two *indica* ecotypes in rice. Theor Appl Genet.

[CR2] Ashraf M (2010). Inducing drought tolerance in plants. Recent advances. Biotechnol Adv.

[CR3] Atlin GN, Lafitte HR, Saxena NP, O’Toole JC (2002). Marker-assisted breeding versus direct selection for drought tolerance in rice. Proceedings of international workshop on field screening for drought tolerance in Rice, India, 2002.

[CR4] Babu RC, Shashidhar HE, Lilley JM, Thanh ND, Ray JD, Sadasivam S, Sarkarang S, O'Toole JC, Nguyen HT (2001). Variation in root penetration ability, osmotic adjustment and dehydration tolerance among accessions of rice adapted to rainfed lowland and upland ecosystems. Plant Breed.

[CR5] Babu RC, Nguyen BD, Chamarerk V, Shanmugasundaram P, Chezhian P, Jeyaprakash P, Ganesh SK, Palchamy A, Sadasivam S, Sarkarung S, Wade LJ, Nguyen HT (2003). Genetic analysis of drought resistance in rice by molecular markers: association between secondary traits and field performance. Crop Sci.

[CR6] Basten CJ, Weir BS, Zeng ZB (2005). QTL Cartographer version 2.5.

[CR7] Bernier J, Kumar A, Ramaiah V, Spaner D, Atlin G (2007). A large-effect QTL for grain yield under reproductive-stage drought stress in upland rice. Crop Sci.

[CR8] Bernier J, Atlin GN, Serraj R, Kumar A, Spaner D (2008). Breeding upland rice for drought resistance. J Sci Food Agric.

[CR9] Bian JM, Jiang L, Liu LL, Wei XJ, Xiao YH, Zhang LJ, Zhao ZG, Zhai HQ, Wan JM (2010). Construction of a new set of rice chromosome segment substitution lines and identification of grain weight and related traits QTLs. Breed Sci.

[CR10] Biji KR, Jeyaprakash P, Ganesh SK, Senthil A, Babu RC (2008). Quantitative trait loci linked to plant production traits in rice under drought stress in a target environment. Sci Asia.

[CR11] Cattivelli L, Rizza F, Badeck FW, Mazzucotelli E, Mastrangelo AM, Francia E, Mare C, Tondelli A, Stanca AM (2008). Drought tolerance improvement in crop plants: an integrated view from breeding to genomics. Field Crop Res.

[CR12] Dixit S, Swamy BM, Vikram P, Ahmed HU, Cruz MS, Amante M, Atri D, Leung H, Kumar A (2012) Fine mapping of QTLs for rice grain yield under drought reveals sub-QTLs conferring a response to variable drought severities. Theor Appl Genet 125:155–169 10.1007/s00122-012-1823-9 22361948

[CR13] Ebana K, Shibaya T, Wu J, Matsubara K, Kanamori H, Yamane H, Yamanouchi U, Mizubayashi T, Kono I, Shomura A, Ito S, Ando T, Hori K, Matsumoto T, Yano M (2011). Uncovering of major genetic factors generating naturally occurring variation in heading date among Asian rice cultivars. Theor Appl Genet.

[CR14] Fehr WR, Fehr WR (1987). Heritability. Principles of cultivar development: theory and technique.

[CR15] Fischer KS, Fukai S, Kumar A, Leung H, Jongdee B (2012). Field phenotyping strategies and breeding for adaptation of rice to drought. Front Physiol.

[CR16] Garrity DP, O’Toole JC (1995). Selection for reproductive stage drought avoidance in rice using infrared thermometry. Agron J.

[CR17] Ghimire KH, Quiatchon LA, Vikram P, Swamy BPM, Hernandez JE, Borromeo TH, Kumar A (2012). Identification and mapping of a QTL with a consistent effect on grain yield under drought. Field Crop Res.

[CR18] Gomez MS, Boopathi NM, Kuma SS, Ramasubramanian T, Chengsong Z, Jeyaprakash P, Senthil A, Babu RC (2010). Molecular mapping and location of QTLs for drought-resistance traits in *indica* rice (*Oryza sativa* L.) lines adapted to target environments. Acta Physiol Plant.

[CR19] Gu J, Yin X, Struik PC, Stomph TJ, Wang H (2011). Using chromosome introgression lines to map quantitative trait loci for photosynthesis parameters in rice (*Oryza sativa* L.) leaves under drought and well-watered field conditions. J Exp Bot.

[CR20] Guo LB, Chu CC, Qian Q (2006). Rice mutants and functional genomics. Chin Bull Bot.

[CR21] Haider Z, Khan AS, Zia S (2012). Correlation and path coefficient analysis of yield components in rice (*Oryza sativa* L.) under simulated drought stress condition. Am-Eurasian J Agric Environ Sci.

[CR22] Hruz T, Laule O, Szabo G, Wessendorp F, Bleuler S, Oertle L, Widmayer P, Gruissem W, Zimmermann P (2008). Genevestigator V3: a reference expression database for the meta-analysis of transcriptomes. Adv Bioinformatics.

[CR23] Hua TH, Wei MH, Qiao YX, Yan XX, Shou LM, Qing ZS, Jun LL (2006). Identification of related QTLs at late developmental stage in rice under two nitrogen levels. Acta Genet Sin.

[CR24] IBM Corp. Released (2012). IBM SPSS statistics for windows, version 21.0.

[CR25] IRRI (International Rice Research Institute) (1996). Standard evaluation system for rice.

[CR26] IRRI (International Rice Research Institute) (2006). Rice breeding course.

[CR27] Jones HG, Serraj R, Loveys BR, Xiong L, Wheaton A, Price AH (2009). Thermal infrared imaging of crop canopies for the remote diagnosis and quantification of plant responses to water stress in the field. Funct Plant Biol.

[CR28] Jongdee B, Fukai S, Cooper M (2002) Leaf water potential and osmotic adjustment as physiological traits to improve drought tolerance in rice. Field Crops Res 76:153–163

[CR29] Kamoshita A, Wade LJ, Ali ML, Pathan MS, Zhang J, Sarkarung S, Nguyen HT (2002). Mapping QTLs for root morphology of a rice population adapted to rainfed lowland conditions. Theor Appl Genet.

[CR30] Kamoshita A, Babu RC, Boopathi N, Fukai S (2008). Phenotypic and genotypic analysis of drought-resistance traits for development of rice cultivars adapted to rainfed environments. Field Crops Res.

[CR31] Kanagaraj P, Prince KSJ, Sheeba JA, Biji KR, Paul SB, Senthil A, Babu RC (2010). Microsatellite markers linked to drought resistance in rice (*Oryza sativa* L.). Curr Sci.

[CR32] Kawahara Y, de la Bastide M, Hamilton JP, Kanamori H, McCombie WR, Ouyang S, Schwartz DC, Tanaka T, Wu J, Zhou S, Childs KL, Davidson RM, Lin H, Quesada-ocampo L, Vaillancourt B, Sakai H, Lee SS, Kim J, Numa H, Itoh T, Buell CR, Matsumoto T (2013). Improvement of the *Oryza sativa*Nipponbare reference genome using next generation sequence and optical map data. Rice.

[CR33] Kumar R, Venuprasad R, Atlin GN (2007). Genetic analysis of rainfed lowland rice drought tolerance under naturally-occurring stress in eastern India: heritability and QTL effects. Field Crops Res.

[CR34] Kumar A, Bernier J, Verulkar S, Lafitte HR, Atlin GN (2008). Breeding for drought tolerance: direct selection for yield, response to selection and use of drought-tolerant donors in upland and lowland adapted populations. Field Crop Res.

[CR35] Lafitte HR, Price AH, Courtois B (2004). Yield response to water deficit in an upland rice mapping population: associations among traits and genetic markers. Theor Appl Genet.

[CR36] Lafitte R, Blum A, Atlin G, Fischer KS, Lafitte R, Fukai S, Atlin G, Hardy B (2004). Using secondary traits to help identify drought tolerant genotypes. Breeding rice for drought-prone environments.

[CR37] Lanceras JC, Pantuwan G, Boonrat J, Toojinda T (2004). Quantitative trait loci associated with drought tolerance at reproductive stage in rice. Plant Physiol.

[CR38] Lenka SK, Katiyar A, Chinnusamy V, Bansal KC (2011). Comparative analysis of drought-responsive transcriptome in Indica rice genotypes with contrasting drought tolerance. Plant Biotechnol J.

[CR39] Li J, Xu P, Deng X, Zhou J, Hu F, Wan J, Tao D (2008). Identification of four genes for stable hybrid sterility and an epistatic QTL from a cross between *Oryza sativa* and *Oryzaglaberrima*. Euphytica.

[CR40] Liu H, Zou G, Liu G, Hu S, Li M, Yu X, Mei H, Luo L (2005). Correlation analysis and QTL identification for canopy temperature, leaf water potential and spikelet fertility in rice under contrasting moisture regimes. Chin Sci Bull.

[CR41] Lopes MS, Reynolds M (2010). Partitioning of assimilates to deeper roots is associated with cooler canopies and increased yield under drought in wheat. Funct Plant Biol.

[CR42] Maccaferri M, Sanguineti MC, Corneti S, Araus Ortega JL, Ben SM, Bort J, DeAmbrogio E, Garcia del Moral LF, Demontis A, El-Ahmed A, Maalouf F, Machlab H, MartosV MM, Motawaj J, Nachit M, Nserallah N, Ouabbou H, Royo C, Slama A, Tuberosa R (2008). Quantitative trait loci for grain yield and adaptation of durum wheat (*Triticum durum*) across a wide range of water availability. Genetics.

[CR43] Manickavelu A, Nadarajan N, Ganesh SK, Gnanamalar RP, Babu RC (2006). Drought tolerance in rice: morphological and molecular genetic consideration. Plant Growth Regul.

[CR44] Manly KF, Cudmore RH, Meer JM (2001) Map Manager QTX, cross-platform software for genetic mapping. Mammalian Genome 12:930–932 10.1007/s00335-001-1016-3 11707780

[CR45] Pandey S, Bhandari H, Ding S, Prapertchob P, Sharan R, Naik D, Taunk SK, Sastri A (2007). Coping with drought in rice farming in Asia: insights from a cross-country comparative study. Agric Econ.

[CR46] Pradeepa N, Priya PS, Prince KSJ, Kavitha S, Poornima R, Prabhakar MS, Babu RC (2012). In Silico analysis of a consensus QTL for drought resistance in rice. Online J Bioinformatics.

[CR47] Richards RA, Rebetzke GJ, Watt M, Condon AG, Spielmeyer W, Dolferus R (2010). Breeding for improved water productivity in temperate cereals: phenotyping, quantitative trait loci, markers and the selection environment. Funct Plant Biol.

[CR48] Salunkhe A, Poornima R, Prince KSJ, Kanagaraj P, Sheeba JA, Amudha K, Suji KK, Senthil A, Babu RC (2011). Fine mapping QTL for drought resistance traits in rice (*Oryza sativa* L.) using bulk segregant analysis. Mol Biotechnol.

[CR49] SAS Institute Inc (1990). SAS user’s guide 1990. Version 6.

[CR50] Sellamuthu R, Liu GF, Serraj R (2011). Genetic analysis and validation of quantitative trait loci associated with reproductive- growth traits and grain yield under drought stress in a double haploid line population of rice (*Oryza sativa* L.). Field Crops Res.

[CR51] Serraj R, McNally KL, Slamet-Loedin I, Kohli A, Haefele SM, Atlin G, Kumar A (2011). Drought resistance improvement in rice: an integrated genetic and resource management strategy. Plant Prot Sci.

[CR52] Srividhya A, Vemireddy LR, Sridhar S, Jayaprada M, Ramanarao PV, Hariprasad AS, Reddy HK, Anuradha G, Siddiq E (2011). Molecular mapping of QTLs for yield and its components under two water supply conditions in rice (*Oryza sativa* L.). J Crop Sci Biotechnol.

[CR53] Steele KA, Gyawali S, Joshi KD, Shrestha P, Sthapit BR, Witcombe JR (2009). Has the introduction of modern rice varieties changed rice genetic diversity in a high- altitude region of Nepal?. Field Crops Res.

[CR54] Suji KK, Prince KSJ, Mankhar PS, Kanagaraj P, Poornima R, Amutha K, Kavitha S, Biji KR, Gomez SM, Chandra Babu R (2012). Evaluation of rice near isogenic lines with root QTLs for plant production and root traits in rainfed target populations of environment. Field Crop Res.

[CR55] Suji KK, Biji KR, Poornima R, Prince KSJ, Amudha K, Kavitha S, Mankar S, Babu RC (2012). Mapping QTLs for plant phenology and production traits using *indica* rice (*Oryza sativa* L.) lines adapted to rainfed environment. Mol Biotechnol.

[CR56] Swamy MBP, Vikram P, Dixit S, Ahmed HU, Kumar A (2011). Meta-analysis of grain yield QTL identified during agricultural drought in grasses showed consensus. BMC Genomics.

[CR57] Tardieu F (2012). Any trait or trait-related allele can confer drought tolerance: just design the right drought scenario. J Exp Bot.

[CR58] Tardieu F, Tuberosa R (2010). Dissection and modelling of abiotic stress tolerance in plants. Curr Opin Plant Biol.

[CR59] Venuprasad R, Lafitte HR, Atlin GN (2007). Response to direct selection for grain yield under drought stress in rice. Crop Sci.

[CR60] Venuprasad R, Sta-Cruz MT, Amante M, Magbanua R, Kumar A, Atlin GN (2008). Response to two cycles of divergent selection for grain yield under drought stress in four rice breeding populations. Field Crops Res.

[CR61] Venuprasad R, Bool ME, Dalid CO, Bernier J, Kumar A, Atlin GN (2009). Genetic loci responding to two cycles of divergent selection for grain yield under drought stress in a rice breeding population. Euphytica.

[CR62] Verulkar SB, Mandal NP, Dwivedi JL, Singh BN, SinhaPK DP, Singh ON, Bose LK, Swain P, Robin S, Chandrababu R, SenthilS JA, Shashidhar HE, Hittalmani S, Vera Cruz C, Paris T, Raman A, Haefele S, Serraj R, Atlin G, Kumar A (2010). Breeding resilient and productive genotypes adapted to drought prone rainfed ecosystems of India. Field Crops Res.

[CR63] Vikram P, Swamy BPM, Dixit S, Ahmed HU, Cruz MTS, Singh AK, Kumar A (2011). qDTY1.1, a major QTL for rice grain yield under reproductive-stage drought stress with a consistent effect in multiple elite genetic backgrounds. BMC Genet.

[CR64] Weber VS, Melchinger AE, Magorokosho C, Makumbi D, Banziger M, Atlin GN (2012). Efficiency of managed-stress screening of elite maize hybrids under drought and low nitrogen for yield under rainfed conditions in South Africa. Crop Sci.

[CR65] Xing YZ, Zhang Q (2010). Genetic and molecular bases of rice yield. Annu Rev Plant Biol.

[CR66] Yadaw BR, Dixit S, Raman A, Mishra KK, Vikram P, Swamy BPM, Cruz Ma TS, Maturan PT, Pandey M, Kumar A (2013). A QTL for high grain yield under lowland drought in the background of popular rice variety Sabitri from Nepal. Field Crops Res.

[CR67] Yang J, Hu C, Hu H, Yu R, Xia Z, Ye X, Zhu J (2008). QTLNetwork: mapping and visualizing genetic architecture of complex traits in experimental populations. Bioinformatics.

[CR68] Ziyomo C, Bernardo R (2013). Drought tolerance in maize: Indirect selection through secondary traits versus genomewide selection. Crop Sci.

