# Supplementary material for: Mapping Consistent Rice (Oryza sativa L.) Yield QTLs under Drought Stress in Target Rainfed Environments
Source: Rice (N Y). 2015 Jul 24;8:25. doi: 10.1186/s12284-015-0053-6 (PMC4513014; doi:10.1186/s12284-015-0053-6)
Supplement: Additional file 1: Table S1. — Mean value of mineral and organic soil moisture contents of irrigated and water stressed field in trial 1. [file 12284_2015_53_MOESM1_ESM.doc]

# Additional Table 1. Mean value of mineral and organic soil moisture contents of irrigated and water stressed field in trial 1

| **Days after imposing stress** | **Mineral soil moisture (m^3^m^-3^)** | | **Per cent reduction under stress** | **Organic soil moisture (m^3^m^-3^)** | | **Per cent reduction under stress** |
| --- | --- | --- | --- | --- | --- | --- |
|  | Control | **Stress** |  | **Control** | **Stress** |  |
| 18 | 0.414 (41.4) | 0.324 (32.4) | 21.1 | 0.482 (48.2) | 0.393 (39.3) | 18.5 |
| 21 | 0.403 (40.3) | 0.303 (30.3) | 24.8 | 0.479 (47.9) | 0.370 (37.0) | 22.8 |
| 26 | 0.390 (39.0) | 0.177 (17.7) | 54.6 | 0.465 (46.5) | 0.232 (23.2) | 50.1 |
| 35 | 0.3659 (35.9) | 0.068 (6.8) | 81.1 | 0.431 (43.1) | 0.114 (11.4) | 73.5 |

Values in parenthesis indicate per cent of mineral and organic soil moisture content.
