# Supplementary material for: Mapping Consistent Rice (Oryza sativa L.) Yield QTLs under Drought Stress in Target Rainfed Environments
Source: Rice (N Y). 2015 Jul 24;8:25. doi: 10.1186/s12284-015-0053-6 (PMC4513014; doi:10.1186/s12284-015-0053-6)
Supplement: Additional file 3: Table S3 — Correlation coefficients among biomass and physio-morphological parameters measured under drought stress in MSE during 2004–2005 (Trial 1). [file 12284_2015_53_MOESM3_ESM.docx]

# Additional Table 3 Correlation coefficients among biomass and physio-morphological parameters measured under drought stress in MSE during 2004-2005 (Trial 1)

| Traits ^a^ | BM | PH | NT | LR | LD | ST | SPAD | CT |
| --- | --- | --- | --- | --- | --- | --- | --- | --- |
| BM | 1 |  |  |  |  |  |  |  |
| PH | 0.334 ^c^ | 1 |  |  |  |  |  |  |
| NT | 0.593 ^c^ | -0.014 | 1 |  |  |  |  |  |
| LR | -0.122 | -0.053 | 0.006 | 1 |  |  |  |  |
| LD | -0.117 | 0.173^b^ | -0.199 ^c^ | 0.576 ^c^ | 1 |  |  |  |
| SR | 0.303^c^ | 0.033 | 0.688 ^c^ | -0.624 ^c^ | -0.483 ^c^ | 1 |  |  |
| SPAD | 0.161 ^b^ | 0.057 | 0.067 | -0.008 | 0.065 | 0.058 | 1 |  |
| CT | -0.317 ^c^ | -0.115 | -0.193 ^c^ | 0.093 | 0.063 | -0.061 | 0.078 | 1 |

^a BM, Biomass (g/m2); PH, Plant height (cm); NOT, number of tiller; LR, Leaf rolling; LD, Leaf drying; SR, Stress recovery; SPAD, SPAD value; CT, Canopy temperature (0C)^

^b p<0.05.^

^c p<0.01.^
