# Supplementary material for: Mapping Consistent Rice (Oryza sativa L.) Yield QTLs under Drought Stress in Target Rainfed Environments
Source: Rice (N Y). 2015 Jul 24;8:25. doi: 10.1186/s12284-015-0053-6 (PMC4513014; doi:10.1186/s12284-015-0053-6)
Supplement: Additional file 4: Table S4 — Correlation coefficients among plant phenology and production traits under rainfed conditions in trial 2 (2004–05) and trial 3 (2009–10) conducted in TPE. [file 12284_2015_53_MOESM4_ESM.docx]

**Additional Table 4**. Correlation coefficients among plant phenology and production traits under rainfed conditions in trial 2 (2004-05) and trial 3 (2009-10) conducted in TPE

| Traits ^a^ | GY | SY | DTF | HSW | HI | NPT | NOT | PH | PHI | PL | SF |
| --- | --- | --- | --- | --- | --- | --- | --- | --- | --- | --- | --- |
| GY  Trial 2  Trial 3 | 1.00 |  |  |  |  |  |  |  |  |  |  |
| SY  Trial 2  Trial 3 | 0.15 ^c^  0.12 ^c^ | 1.00 |  |  |  |  |  |  |  |  |  |
| DTF  Trial 2 | - 0.51 ^c^ | 0.54 ^c^ |  |  |  |  |  |  |  |  |  |
| HSW  Trial 2 | 0.03 | -0.07 | -0.07 | 1.00 |  |  |  |  |  |  |  |
| HI  Trial 2  Trial 3 | 0.59 ^c^  0.59 ^c^ | - 0.64 ^c^  - 0.66 ^c^ | - 0.84 ^c^  - | 0.12 ^b^  - | 1.00 |  |  |  |  |  |  |
| NPT  Trial 2  Trial 3 | 0.07  0.11 ^b^ | - 0.08  0.12 ^b^ | - 0.17 ^c^  - | 0.06  - | 0.14 ^b^  - 0.01 | 1.00 |  |  |  |  |  |
| NOT  Trial 2  Trial 3 | - 0.01  0.11 ^b^ | -0.12 ^b^  0.10 | - 0.10 - | 0.02  - | 0.09  0.00 | 0.66 ^c^  0.97 ^c^ | 1.00 |  |  |  |  |
| PH  Trial 2  Trial 3 | 0.51 ^c^  0.39 ^c^ | 0.43 ^c^  0.34 ^c^ | - 0.13 ^b^  - | - 0.05  - | 0.02  0.01 | - 0.11 ^b^  0.15 ^c^ | - 0.13 ^b^  0.14 ^c^ | 1.00 |  |  |  |
| PHI  Trial 2 | 0.47 ^c^ | -0.06 | - 0.06 | 0.05 | 0.63 ^c^ | 0.02 | 0.02 | 0.02 | 1.00 |  |  |
| PL  Trial 2  Trial 3 | 0.38 ^c^  - 0.03 | 0.37 ^c^  0.44 ^c^ | - 0.04  - | - 0.04  - | - 0.05  - 0.39 ^c^ | - 0.10  0.09 | - 0.13 ^b^  0.08 | 0.77 ^c^  0.39 ^c^ | 0.14 ^b^  - | 1.00 |  |
| SF  Trial 3 | 0.35 ^c^ | - 0.19 ^c^ | - | - | 0.41 ^c^ | - 0.04 | - 0.06 | 0.21 ^c^ | - | - 0.08 | 1.00 |

^a GY, Grain yield (g/m2); SY, Straw yield (g/m2); SPY, Single plant yield (g/m2); DTF, Days to fifty per cent flowering (days); HSE, hundred seed weight (g); HI, Harvest index; NPT, No of productive tillers; NOT, No of tillers; PH, Plant height (cm); PHI, Panicle harvest index; PL, Length of panicle (cm); SF, Spikelet fertility (%). (‘-‘denotes the trait not measured in the respective trials)^

^b p<0.05.^

^c p<0.01.^
